# Reporting of scar outcomes in the hand and wrist; a state-of-the-art literature review

**DOI:** 10.1186/s12891-023-06296-y

**Published:** 2023-03-31

**Authors:** Donna L. Kennedy, Tracy Chism-Balangue, Dominic Furniss

**Affiliations:** 1 Therapy Department, Charing Cross Hospital, Fulham Palace Road, London, W6 8RF UK; 2 Pain Research, Department of Surgery and Cancer, Imperial College London, London, UK; 3 Occupational Therapy, West Coast University, Los Angeles, CA USA; 4 Nuffield Department of Orthopaedics, Rheumatology, and Musculoskeletal Science, University of Oxford, Botnar Research Centre, Oxford, UK

**Keywords:** Scar, Outcome measure, Pain, Hypersensitivity, Appearance, Psychological

## Abstract

**Objectives:**

The aim of this literature review was to synthesise and report current practice in evaluation and reporting of scar outcomes in hand and wrist clinical research.

**Methods:**

A systematic search from inception to 2022 was conducted using three electronic databases. English language randomized controlled trials and observational cohort studies reporting standardised scar outcome measures and/or scar symptoms, appearance, impairment, function, or mental health outcomes in patients with hand and wrist scars were included. Two independent reviewers determined study eligibility and performed data extraction of a priori identified scar outcome domains. Data analysis included descriptive statistics and identification of discordance in taxonomy.

**Results:**

Fifty-nine studies were included. Elective surgery cohorts were the most frequently included clinical population (*n* = 28; 47%) followed by burns (*n* = 16; 27%). Six different standardised scar outcome measures were reported by 25% of studies however only 7% of studies utilised a patient-reported measure. Scar symptoms were the most frequently reported outcome domain (81%); but taxonomy was incongruous, constructs lacked working definitions required for generalisability and outcome measurement was variable and unreported. Nineteen different measures of scar appearance and structure were reported by 30 (51%) of studies however only nine (23%) were patient-reported. Seven different hand function PROMs were reported by 25 (43%) studies. Person-centred domains including scar acceptability (12%), mental health impact (5%), and social participation (4%) were rarely reported.

**Conclusions:**

This review highlights that evaluation and reporting of hand and wrist scar outcomes is not standardised, assessment methods and measures are under-reported and there is discordance in taxonomy. Evaluation is not person-centred, rather it is dependent on clinician assessment. Domains including scar acceptability, mental health, and social participation are rarely addressed. A stakeholder consensus derived hand and wrist scar core outcome measurement set will promote standardisation and underpin improvements in clinical research quality, transparency, and rigour.

**Supplementary Information:**

The online version contains supplementary material available at 10.1186/s12891-023-06296-y.

## Background

Scars in the hand and wrist, whether secondary to traumatic injury or planned surgery, are common and burdensome for patients. In the United Kingdom [1] and the United States [2], approximately twenty percent of patients attending Accident & Emergency present with injuries to the hand and wrist. While not all traumatic hand injury will result in a scar, analysis of Hospital Episode Statistics (HES) in England demonstrates that more than 50% of hand trauma patients present with a wound or laceration, inevitably resulting in scar [3]. Likewise, hand scars are an unavoidable consequence of surgical treatment for pathologic or acquired hand conditions. Following planned hand surgery, up to 50% of patients report scar hyperesthesia and/or functional interference, which can persist at 2 years post-surgery [4–7]. The significant burden posed by hand scarring was recently highlighted by a British Society for Surgery of the Hand—James Lind Alliance Priority Setting Partnership, where treatment to improve scar and fibrosis formation following hand surgery or trauma was identified as a top ten research priority [8].

Scar pain and hypersensitivity, though commonly reported adverse events, have no diagnostic criteria [9, 10] and evaluation techniques are not standardised [11–13]. Although the physical characteristics of scar are thought to be related to hypersensitivity, there is no evidence for how the morphology of a hypersensitive scar differs from a quiescent scar [14, 15]. Persistent scar pain may be related to the extent of local tissue trauma and/or associated with psychological factors, but the underlying mechanisms are unclear and likely multifactorial [16].

While there is emerging evidence for the psychosocial impact of scars and the detrimental effect that scars may have on quality of life, this scar burden receives little attention in the literature. In “The hidden cost of skin scars; quality of life after skin scarring”, Brown et al. (2008) explored the effects of scarring. The authors report significant, multidimensional impacts of scarring; patients report feeling stigmatised, anxious, and angry because of their scars. Patients report that hand scars impact their personal and work lives and emotional well-being [17]. This work suggests the multidimensional evaluation of hand scars is crucial to capture the effects of scarring that are important to patients.

Recommendations for scar evaluation suggest a comprehensive patient-centred evaluation should assess physical characteristics, cosmetic appearance, and symptoms including the impact of scar on activity, social participation, and quality of life [18]. However, while there are numerous available scar evaluation tools, there is no universally accepted clinician-completed or patient-reported outcome measure (PROM) [12]. The first standardised scar evaluation measure, the Vancouver Scar Scale (1990) was a clinician-completed evaluation of scar physical qualities [19]. Over the past three decades, numerous scar assessment measures have been developed. In 2004, the first tool to innovatively include both the clinician *and* patient assessment of scar, the Patient and Observer Scar Assessment scale (POSAS), was developed [20]. Importantly, POSAS domains included patient evaluated scar physical symptoms, appearance, and global rating of scar. Further progress in patient-centred scar evaluation was made in 2009 with the development of the Patient Scar Assessment Questionnaire (PSAQ) [21], a patient completed assessment of scar appearance, symptoms, and scar consciousness. In an evaluation of scar assessment methods, Lipman et al. (2020) provide a comprehensive overview of the evolution of current scar evaluation tools. Despite advances in scar evaluation, the authors conclude that current tools fail to fully capture the impact of scarring on patients’ function, psychosocial health, and quality of life [22].

The impact of scarring is multidimensional; therefore, it may be unrealistic to expect that one scar evaluation measure can adequately capture the numerous outcome domains of importance to people with scars. However, at present, a consensus-derived core outcome set [23] for the evaluation of hand and wrist scars is lacking. Given the lack of concurrence in scar evaluation, a review exploring the evaluation and reporting of scar outcomes in the hand and wrist provides important insight and is warranted. This research aimed to explore the state-of-the-art of current practice in evaluation and reporting of scar outcomes in hand and wrist clinical research, identify discordance in taxonomy and highlight domains for inclusion in future scar reporting consensus activities [24].

## Methods

A study design of ‘state-of-the-art’ literature review was used to provide a synthesis of current practice and identify priorities for future research, as opposed to identifying treatment uncertainties or making recommendations for care [25]. Systematic methods were employed, however, as the review objective was to generate a descriptive summary of current reporting practice, critical appraisal of risk of bias of the included literature would have been inappropriate and was therefore omitted. PRISMA guidelines for reporting of systematic reviews were followed [26]. The review protocol was registered on Open Science Framework https://osf.io/74an6/.

A search strategy (Supplementary data 1) was developed and piloted by the review team with the guidance of an NHS Support Librarian, Imperial College London. Medline, Embase and CINAHL databases were initially searched from inception until May 2020. The literature search was thereafter updated to locate additional reports published until 2^nd^ December 2022. English language, full primary research reports (randomised controlled trials [RCTs] and observational studies [cohort and repeated methods]) in humans were included. There were no age exclusions. Evidence syntheses, abstracts and conference proceedings, case reports, case series, study protocols, narrative reviews, and non-English language papers were excluded.

Two reviewers (DLK; TCB) independently performed title and abstract screening and any disagreements were discussed and taken to a third reviewer (DF) if consensus was not reached. Following the initial screening, full text manuscripts were screened against review inclusion and exclusion criteria. Two reviewers independently performed data extraction using a piloted proforma (Supplementary data 2). Study participant characteristics including gender, age (child or adult), clinical condition, and procedure or intervention were recorded. Study characteristics including year of publication, timepoint of scar assessment, scar evaluation domains, and relevant outcome measures were extracted. Data synthesis included the generation of descriptive statistics and identification of variability in taxonomy pertaining to scar outcomes.

## Results

### Study characteristics

**Fig. 1:**
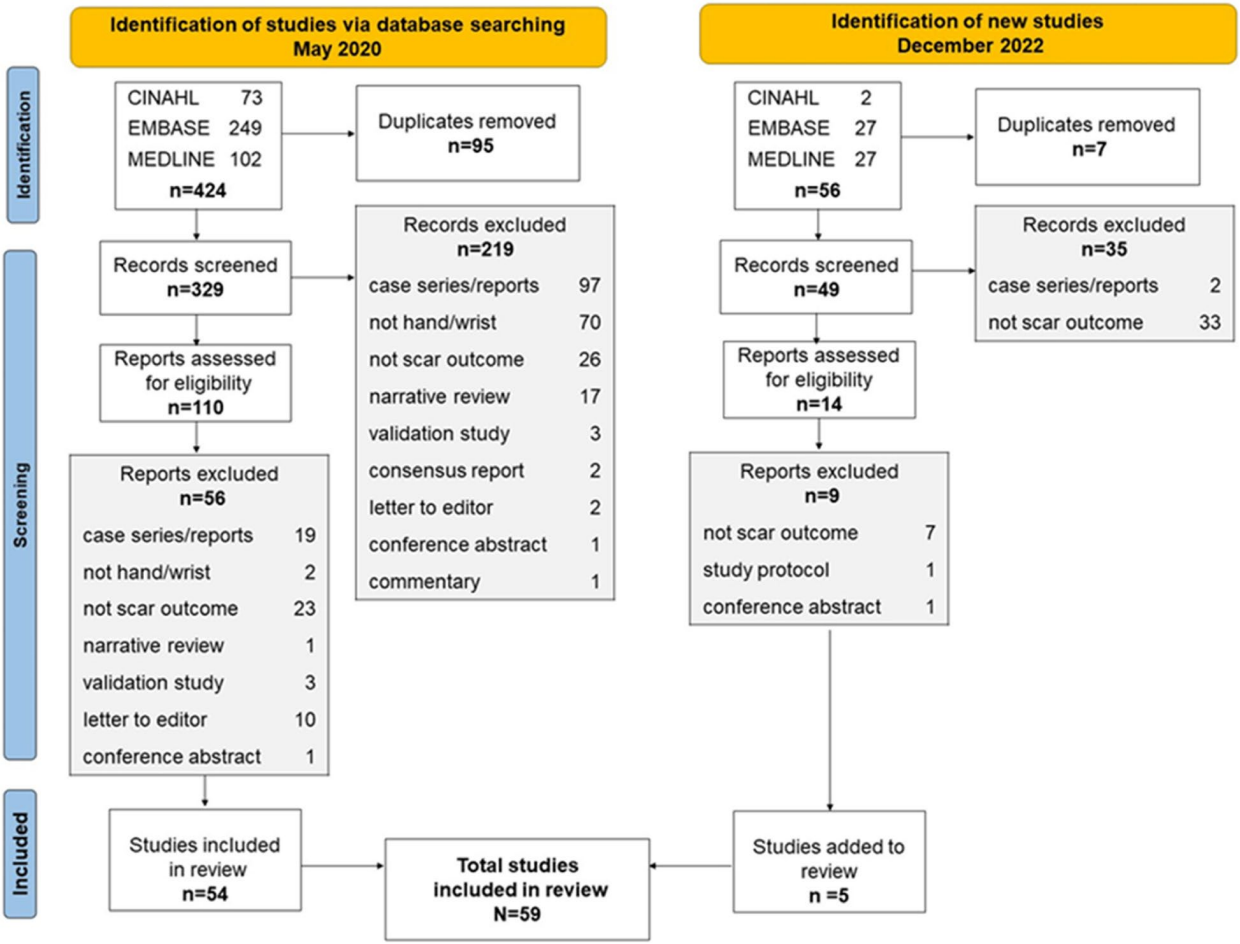
Literature search and screening flow diagram

**Fig. 2:**
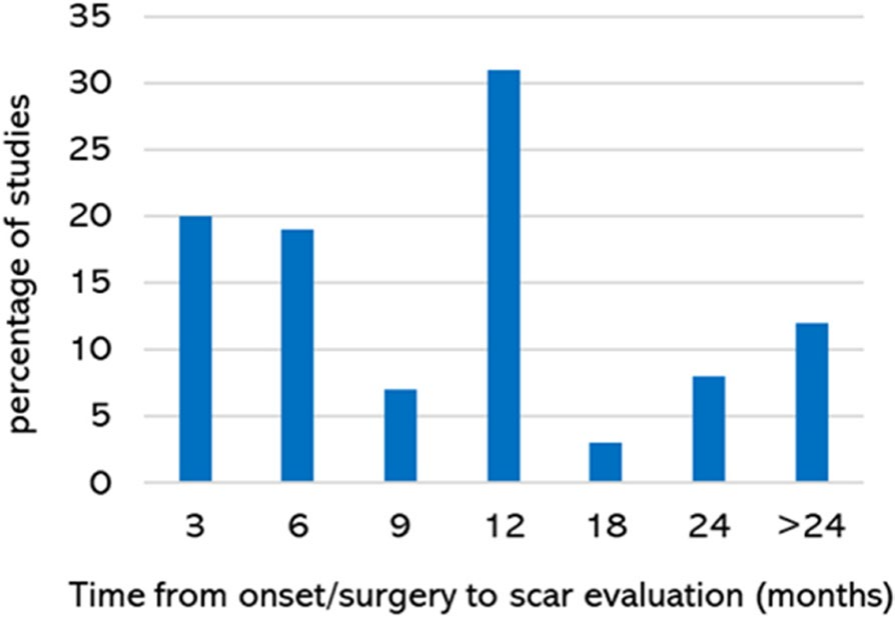
Time from onset to scar evaluation (in months)

Results of the literature search and each stage of literature screening are reported in Fig. 1. A total of 424 records were identified in the initial search, of which 54 were included. An additional 56 records were identified in the search update, with five reports included for a total of 59 reports included in the amalgamated synthesis. Design parameters, reported scar outcome domains, and relevant assessment tools for included studies are reported in Supplementary data 3. Included studies were RCTs [*n* = 22] and cohort studies [*n* = 37]. Publication dates ranged from 1993 to 2022. Data collection was retrospective in ten studies and prospective in 49 studies. Study outcomes were reported at multiple time points, with 20% of studies evaluating outcomes at three months; 31% at one year and 20% at two years or longer (Fig. 2). The plan for the evaluation of scar outcomes was reported in the study methods in 51 (86%) studies. Scar evaluation was by patient report in ten (17%) studies; patient *and* clinician assessment in 34 (58%) studies; clinician assessment in fourteen (24%) studies and by a carer in one (2%) study.

### Participants

The fifty-nine studies comprised 5972 participants: [RCTs *n* = 1748; cohort studies *n* = 4224]. Elective surgery cohorts *n* = 28 (47%) were the most frequently included clinical population [carpal tunnel release *n* = 18; Dupuytren’s excision *n* = 3; trigger finger/thumb *n* = 3; basal thumb joint osteoarthritis *n* = 2; ganglion excision *n* = 1; Dequervain’s release *n* = 1] followed by patients with burns *n* = 16 (27%). Less frequently, studies were conducted in patients following trauma or fracture (*n* = 8; 14%); patients undergoing treatment of scar (*n* = 2; 3%); patients undergoing skin grafting secondary to skin cancer (*n* = 2; 3%) and patients with congenital hand conditions (*n* = 2; 3%). One study (2%) investigated scar outcomes in a cohort comprising patients with scarring secondary to burns, traumatic injury, and surgical scarring. Six studies reported scar outcomes in children, six studies included both children and adults and forty-seven included adults. One study investigating arthroplasty for basal thumb joint arthritis included females only [27], all other studies included both males and females.

### Standardized scar outcome measures

A standardised scar outcome measure was included in 15 (25%) studies (Table 1); with three (5%) studies including two standardised scar assessment tools [28–30]. Only five (8%) studies utilised a patient-reported standardised scar outcome measure (PROM).

**Table 1:** Standardised scar outcome measures utilised in the evaluation of patients with hand and wrist scars

Measure	*n* =	Rater	Domains	Cited by
Vancouver Scar Scale [19]	11	clinician	Pigmentation, pliability, height & vascularity	[28–38]
Patient & Observer Scar Assessment Scale (POSAS) [20]	3	clinician & patient	Patient: pain, itching, colour, stiffness, thickness & relief. Observer: vascularity, pigmentation, thickness, relief, pliability & surface area	[29, 30, 39]
Silverberg Scar Mobility Rating Scale [40]	1	clinician	Range of motion, scar pliability & vascularity	[41]
Matching Assessment of Scars and Photographs (MAPS) [42]	1	clinician	Border height, thickness, colour/pigmentation, surface & localization	[43]
University of North Carolina Scar Scale (UNC4P) [28]	1	patient	Pain, paraesthesia, pliability, pruritis	[28]
SCAR-Q [44]	1	patient	Appearance, symptoms, psychosocial impact	[45]

### Scar physical symptoms

Physical symptoms were the most frequently reported outcome domain (81% of studies); however, taxonomy and outcome measurement were variable (Table 2). Pain was reported as a physical symptom in 20 (34%) studies and quantified or qualified using PROMS or visual and verbal rating scales, however five of the 24 studies did not elaborate on the method of pain evaluation. Scar sensitivity or hypersensitivity was reported in nine studies; however, a working definition of scar hypersensitivity was not provided. Scar tenderness was reported in eight studies; again, it is unclear how tenderness was defined and whether scar tenderness is a construct independent of hypersensitivity. Seven studies reported itch as a physical symptom. Finally, one study reported scar comfort, but a working definition of the construct was not provided.

**Table 2:** Taxonomy of scar physical symptoms reported in the evaluation of patients with hand and wrist scars

Descriptor	Number of studies	Measure (cited by)
Pain	20	◾ VAS [46–50] ◾ POSAS [29, 30, 39] ◾ BCTQ [49, 51] ◾ DASH [34, 52] ◾ Likert scale [51, 53] ◾ PEM [54] ◾ MHQ [55] ◾ UNC4P [28] ◾ Verbal report [56] ◾ Modified Würzburg Wound Score [57] ◾ SCAR-Q [45] ◾ Not reported [27, 58–61]
Sensitivity/hypersensitivity	12	◾ Subjective report [62–64] ◾ Subjective complaint [65] ◾ Pressure algometry [66–68] ◾ SCAR-Q [45] ◾ Not reported [69–71]
Tenderness	8	◾ Present/absent [72, 73] ◾ Likert scale [31, 74] ◾ Not implemented [75] ◾ Not reported [58, 61, 76]
Itching	7	◾ POSAS [29, 30, 39] ◾ Itch Severity Scale [77] ◾ SCAR-Q [45] ◾ Likert scale [53] ◾ Not reported [33]
Comfort	1	◾ VAS [78]

*BCTQ* Boston Carpal Tunnel Questionnaire [79], *DASH* Disabilities of the arm, shoulder and hand [80], Itch Severity Scale [81], *MHQ* Michigan Hand Assessment Questionnaire [82], *POSAS* Patient and Observer Scar Assessment Scale [20], *PEM* Patient Evaluation Measure [83], SCAR-Q [44], *UNC4P* University of North Carolina Scar Scale [28], *VAS* visual analogue scale

### Scar appearance & physical structure

Nineteen different measures of scar appearance and structure, in various constructs, were reported by 30 (51%) studies (Table 3). Only nine of 39 (23%) physical appearance measures were patient-reported. Scar appearance was evaluated with four different standardised outcome measures which incorporated varying appearance constructs of interest; the clinician completed Vancouver Scar Scale (VSS) was the most frequently reported standardised scar appearance assessment measure (*n* = 11) studies. The constructs of scar appearance [57, 75, 84]; cosmesis [45, 56, 85] and aesthetic outcome [38, 75] were reported in six studies. It is unclear however, given the discordance in taxonomy, whether a common scar domain was being evaluated and reported.

**Table 3:** Standardised measures and taxonomy utilised in the evaluation of hand and wrist scar appearance & physical structure

Measure/ Descriptor	Rater	Domains / Constructs (cited by)
Vancouver Scar Scale [19]	Clinician	Pigmentation, pliability, height & vascularity [28–38]
Patient & Observer Scar Assessment Scale [20]	Patient & Clinician	Patient: colour, stiffness, thickness & relief Observer: vascularity, pigmentation, thickness, relief, pliability & surface area [29, 30, 39]
Matching Assessment of Scars & Photographs [42]	Clinician	Border height, thickness, colour & surface texture [43]
SCAR-Q [44]	Patient	Length, width, colour, shape, size & appearance up close and from different angles [45]
Appearance	Patient Clinician	◾ Likert scale [56, 84] ◾ VAS (extremely ugly to perfectly normal) [75]
Cosmesis	Patient	◾ Cosmesis scale MHQ [56] ◾ Satisfaction; modified Würzburg Wound Score [57] ◾ Not reported [85]
Aesthetic outcome	Clinician	◾ VAS [38] ◾ VAS (extremely ugly to perfectly normal) [75]
Colour	Clinician	VAS [86]
Pigmentation	Clinician	VAS [78]
Vascularity	Clinician	VAS [78]
Height	Clinician	◾ Ruler (millimetres) [84] ◾ Water displacement [54]
Thickness	Clinician	◾ VAS [86]
Adherence	Clinician	Not reported [87]
Pliability/Mobility	Clinician	◾ Skin glide grade scale [41] ◾ VSS pliability scale [88] ◾ Cutometer [89]
Softness	Clinician	VAS [86]
Hypertrophy	Clinician	Present or absent [73]
Volume	Clinician	Ultrasound [90]
Length	Clinician	Centimetres [65]
Hair growth	Clinician	Present or absent [31]

*MHQ* Michigan Hand Assessment Questionnaire [82], *VAS* visual analogue scale

### Hand function assessment

Standardised patient-reported outcome measures (PROMs) of hand function were reported in 23 (39%) of studies, with three studies reporting both a disease specific and generic hand function outcome measure [48, 52, 76]. The Disabilities of the Arm, Shoulder and Hand Outcome Measure (DASH) [80], was the most frequently implemented hand function PROM, reported in 12 (20%) of studies. The Boston Carpal Tunnel Questionnaire, a disease-specific measure of self-reported symptom severity & functional status [79], was reported in six studies. The Michigan Hand Questionnaire (MHQ), evaluating unilateral & bilateral hand function, pain, work performance, aesthetics & satisfaction, was reported by four studies. The QuickDASH [91] and the Patient Evaluation Measure (PEM) [83], comprising the components of the patients’ opinion on the delivery of care, hand health profile & overall assessment, were both reported by two studies. The Patient-rated Wrist and Hand Evaluation (PRWHE) [92] was reported by one study. Finally, the Canadian Occupational Performance Measure (COPM) [93], including outcomes of self-care, productivity & leisure, was reported by one study.

In addition to patient reported measures of function, impairment measures were commonly reported. Hand grip strength, as assessed with hand dynamometry, was the most frequently reported measure of hand impairment (*n* = 22 [37%]). Range of motion, as assessed with goniometry, was reported by 15 (25%) studies. Range of motion assessment and reporting varied, based on the clinical cohort and research question of interest. For example, in an elective surgery cohort of patients undergoing Dupuytren’s surgery, total active motion of the affected digits was reported [88]. In contrast, in patients undergoing thumb reconstruction, range of motion of the individual thumb joints was reported [75].

Sensory function was reported by nine (15%) of studies, including the use of Semmes–Weinstein monofilaments [66–68, 88] and/or two-point discrimination [33, 38, 45, 63, 64, 66–68]. Sensory testing protocols varied, with testing conducted in the area of scarring in some cohorts [33, 38, 45] and within the sensory distribution of the affected digits in others [66, 68, 88]. Rarely, standardised measures of hand functional dexterity, including the Purdue Pegboard (Tiffin and Asher [94]) [68]; Jebsen-Taylor Hand function Test (Jebsen et al. [95]) [68] and Carroll Upper Limb Functional Evaluation (Carroll [96]) [36] were reported.

### Scar acceptability / patient satisfaction

Measures of scar acceptability or satisfaction with scar outcome were included in seven (12%) studies. One study reported patient-rated acceptability using a visual analogue scale [78]. Satisfaction with outcome was assessed using willingness to pay as a surrogate measure [28]; satisfaction with appearance as a proxy measure [84]; and with patient-completed Likert scales [53, 97]. In one study, patient satisfaction was physician-rated [98] and in one study the methods for ascertaining satisfaction were not reported [33].

### Mental health impact & participation restriction

The impact of scarring in the hand or wrist on patients’ mental health was included by three studies. In paediatric patients with burns, self-esteem was evaluated using the Piers-Harris Children’s Concept Scale [99] by Abdullah et al. [100]. Psychological function was evaluated [36] with the relevant scale of the abbreviated Burn Specific Health Scale [101] and psychosocial impact [46] was evaluated with the relevant SCAR-Q scale [44]. Participation restriction secondary to scarring and/or the participants underlying clinical condition was evaluated by two (3%) studies. In patients undergoing fasciectomy for Dupuytren’s disease, Engstrand et al. [88] evaluated patient-reported safety and social issues including concern about the appearance of the hand and avoiding use of the hand in social contexts with a 10-point visual analogue scale. In patients with traumatic fingertip injuries Schultz et al. [45] evaluated use of the hand in everyday life using a binary rating scale.

## Discussion

This state-of-the-art literature review was undertaken to identify, synthesise and report on current methods for evaluation and reporting of scar outcomes in hand and wrist clinical research, whilst identifying discordance in taxonomy. Additionally, it was aimed to identify relevant scar associated domains for inclusion in future scar reporting consensus activities. Fifty-nine reports, published between 1993 and 2022, were included in the synthesis.

We identified that hand scar evaluation and reporting lack standardisation essential for evidence synthesis. There is limited use of standardised scar outcome measures; where standardised measures are utilised, there is variation. There is disparity and inconsistency in outcome domains included in hand scar evaluation. There is discordance in relevant taxonomy and outcome measurement of scar symptoms, appearance, and physical structure. Hand scar evaluation is not person-centred, as patient-reported outcomes are seldom utilised and domains including scar acceptability, mental health impact, and social participation are rarely reported. We identified the following scar associated domains relevant for inclusion in future scar reporting consensus activities: physical symptoms, appearance, physical structure, functional impairment, participation restriction, mental health impact, acceptability, and satisfaction.

Six different standardised scar outcome measures were utilised by fifteen of the fifty-nine included hand and wrist clinical research studies; the Vancouver Scar Scale [19], the Patient and Observer Scar Assessment Scale (POSAS) [20], the Silverberg Scar Mobility Rating Scale [40], Matching Assessment of Scars and Photographs (MAPS) [42], the University of North Carolina Scar Scale (UNC4P) [28] and SCAR-Q [44]. It is unclear why only 25% of studies included a standardised scar outcome measure; it may be that scar outcomes were not identified as a study priority or perhaps there was no available measure deemed fit for purpose at the time of the investigation.

Physical symptoms secondary to scar was the most frequently reported scar outcome domain. However, taxonomy for symptoms was variable and included pain, sensitivity, hypersensitivity, and comfort, without relevant working definitions. Furthermore, within each reported symptom parameter, this was discordance in the method of outcome evaluation. While pain was reported by one third of included studies, assessment predominantly focused on pain intensity. Important persistent pain parameters, including pain interference, frequency, nature, and quality were under-reported [102]. A more robust and uniform approach to the evaluation of scar symptoms will underpin improvements in scar clinical trials and may support the elucidation of mechanisms driving persistent scar pain.

Scar sensitivity, hypersensitivity, or tenderness, while commonly reported, lack working definitions as required to ensure conformity and consistency in assessment. The International Association for the Study of Pain (IASP [103]) define hyperesthesia as an increased sensitivity to stimulation, including touch and thermal stimuli, that may or may not be painful. As such, hyperesthesia includes both allodynia and hyperalgesia and may in fact be the working definition of scar hypersensitivity. Standardising taxonomy for the description of an individuals’ scar sensory experience, or physical symptoms secondary to scar, is required to support future evidence synthesis.

The evaluation of scar physical structure or morphology, as distinct from scar cosmetic appearance, lacks distinction. While it might be presumed that the patient is the best judge of the appearance of their scar, this review identified that only 23% of included scar appearance measures were patient-reported. The patient *AND* observer completed POSAS [20] was reported by three studies. However, the patient-completed POSAS domains are specific to scar structure and symptoms and do not include a patient-derived assessment of scar cosmesis. Importantly, it has also been reported for the POSAS that patient and observer ratings do not concur; patient ratings are poorer than those of clinicians, highlighting the imperative of patient reported scar outcomes [12]. SCAR-Q, developed in 2018 [44], was reported by one study [46]. The SCAR-Q appearance scale quantifies how much a patient is bothered by the appearance of their scar, including scar length, width, colour, shape, and size. The distinction between patient-rated scar appearance and the bothersomeness of appearance may be an important one, as it might be expected that those who are more bothered will possibly report lower quality of life and be more apt to seek secondary scar modification treatments.

We identified seven standardised hand function PROMs, reported by 39% of studies. In addition, impairment measures such as hand grip strength were reported by 37% of studies, range of motion (25%), sensory function (15%) and dexterity (5%). While these measures are specific to function and impairment of the hand and were not implemented to capture the functional impact of scar per se, clearly scarring in the hand and wrist can be deleterious to hand function. Whereas consensus group work to identify outcomes for the evaluation of hand function after burn injuries proposed hand function be assessed with the *Quick*DASH PROM [91] it is not clear how this decision was derived [104]. Furthermore, although the *Quick*DASH is widely used internationally, there are questions as to the robustness of the evidence supporting the measure’s psychometric properties [105–107]. At present, there is no evidence synthesis evaluating the clinical relevance, reliability, or responsiveness of hand function PROMs or hand impairment measures in a clinical population with hand and wrist scars; this clearly warrants further investigation.

Previous qualitative research in patients with scarring in the hand and wrist identified scar acceptability and impact on mental health and social function as important scar outcome domains [17]. However, these domains received scant attention in the literature included in the present review. Scar acceptability or satisfaction with scarring was reported by only 12% of studies. Of the 59 included studies, only three studies reported scar impact on mental health and two on social function. There is growing evidence that patients report detrimental mental health effects and impaired social participation secondary to scarring in the hand and wrist [108]. To mediate for the negative impact of scarring and improve quality of life, patients commonly seek out camouflaging treatments [109–111]. Whereas validated patient-self report measures of adjustment to issues of appearance, such as the Derriford Appearance Scale (DAS24) [112] are available, such measures are not widely implemented in patients with scarring in the hand and wrist. Further research is required to assess the psychometric properties, clinical feasibility, and patient-rated acceptability of such measures.

Previous reviews have evaluated the feasibility and psychometric properties [12] and clinical relevance [22] of available scar outcome measurement tools. This review adds a descriptive synthesis of current practice in scar evaluation and reporting in hand and wrist clinical research. It is anticipated this work will highlight the substantial discordance between the patient reported impact of scarring [17] and current methods of scar evaluation and reporting in hand and wrist clinical research, thereby promoting a more patient-centered approach in future studies. Importantly, the findings of this review underpin the need for a shared taxonomy and standardisation in hand and wrist scar assessment as required for future evidence synthesis.

This state-of the-art literature review took a systematic approach to reviewing the scar evaluation and reporting literature. Search strategy was developed with the support of an information specialist and double-reviewer screening and extraction were employed. For transparency, the full data set is included as supplemental data. Nonetheless, several design weaknesses require consideration. Given the broad search inclusion criteria, the review includes a vast number of clinical cohorts, as well as children and adults. Therefore, and importantly, evaluation and reporting practice in particular cohorts cannot be discerned. Comprehensive demographics including ethnicity and socioeconomic status were not reported for the included clinical cohorts, therefore the generalisability of our findings is impeded. Lastly, we did not report the country where studies were conducted, so possible geographical differences in practice cannot be detected.

## Conclusion

The evaluation and reporting of hand and wrist scar outcomes is not standardised, there is under-reporting of assessment methods, and there is significant discordance in taxonomy which hinders evidence synthesis. Outcome evaluation is not patient-centred but rather is dependent on clinician assessment. Domains including scar acceptability and the impact of scarring on quality of life are rarely addressed. A consensus, stakeholder (patients, clinicians, researchers) derived hand and wrist scar core outcome measurement set will promote standardisation, underpin improvements in clinical research quality, transparency, and rigour, and provide a solid basis for advances in evidence-based treatment.

## Supplementary Information

**Additional file 1.** Search strategy.

**Additional file 2 **

**Additional file 3 **

**Additional file 4 **

## Data Availability

The study data extraction sheet and all extracted data have been shared as supplementary material.

## Abbreviations

BCTQ: Boston Carpal Tunnel Questionnaire
COPM: Canadian Occupational Performance Measure
DASH: Disabilities of the arm, shoulder and hand
MAPS: Matching Assessment of Scars and Photographs
MHQ: Michigan Hand Assessment Questionnaire
PEM: Patient Evaluation Measure
PROMs: Patient reported outcome measures
POSAS: Patient and Observer Scar Assessment Scale
PRWHE: Patient-rated Wrist and Hand Evaluation
RCT: Randomised controlled trial
UNC4P: University of North Carolina Scar Scale
VAS: Visual analogue scale
