# Supplementary material for: Reporting of scar outcomes in the hand and wrist; a state-of-the-art literature review
Source: BMC Musculoskelet Disord. 2023 Mar 31;24:249. doi: 10.1186/s12891-023-06296-y (PMC10064537; doi:10.1186/s12891-023-06296-y)
Supplement: Supplementary file 1 — Additional file 1. Search strategy. [file 12891_2023_6296_MOESM1_ESM.docx]

SUPPLEMENTARY DATA 1_Search Strategy

**Medline**

**Population:**

Hand/ or Hand.mp.

wrist.mp. or Wrist/ or Wrist Joint/

AND

**Intervention:**

scar.mp. or Cicatrix/

injury.mp. or “Wounds and Injuries”/

trauma.mp. or “Wounds and Injuries”/

surgery.mp. or General Surgery/

procedure.mp or Methods/

dermatology.mp or Dermatology/

AND

**Outcomes:**

Patient Reported Outcome Measures/ or Treatment Outcome/ Outcome Assessment, Health Care/ or “Outcome and Process Assessment, Health Care/ or outcome.mp or Patient Outcome Assessment/

satisfaction.mp. or Personal Satisfaction/

pain.mp. or Pain Measurement or Chronic Pain/ or Nociceptive Pain/ or pain/ or pain perception/ or Complex Regional Pain Syndrome/

activities of daily living.mp. or “Activities of Daily Living”/

mood.mp.
